# Supplementary material for: Geographic distribution of nematodes in the Atacama is associated with elevation, climate gradients and parthenogenesis
Source: Nat Commun. 2026 Jan 9;17:424. doi: 10.1038/s41467-025-67117-5 (PMC12796448; doi:10.1038/s41467-025-67117-5)
Supplement: Supplementary file 1 — Supplementary Information [file 41467_2025_67117_MOESM1_ESM.pdf]

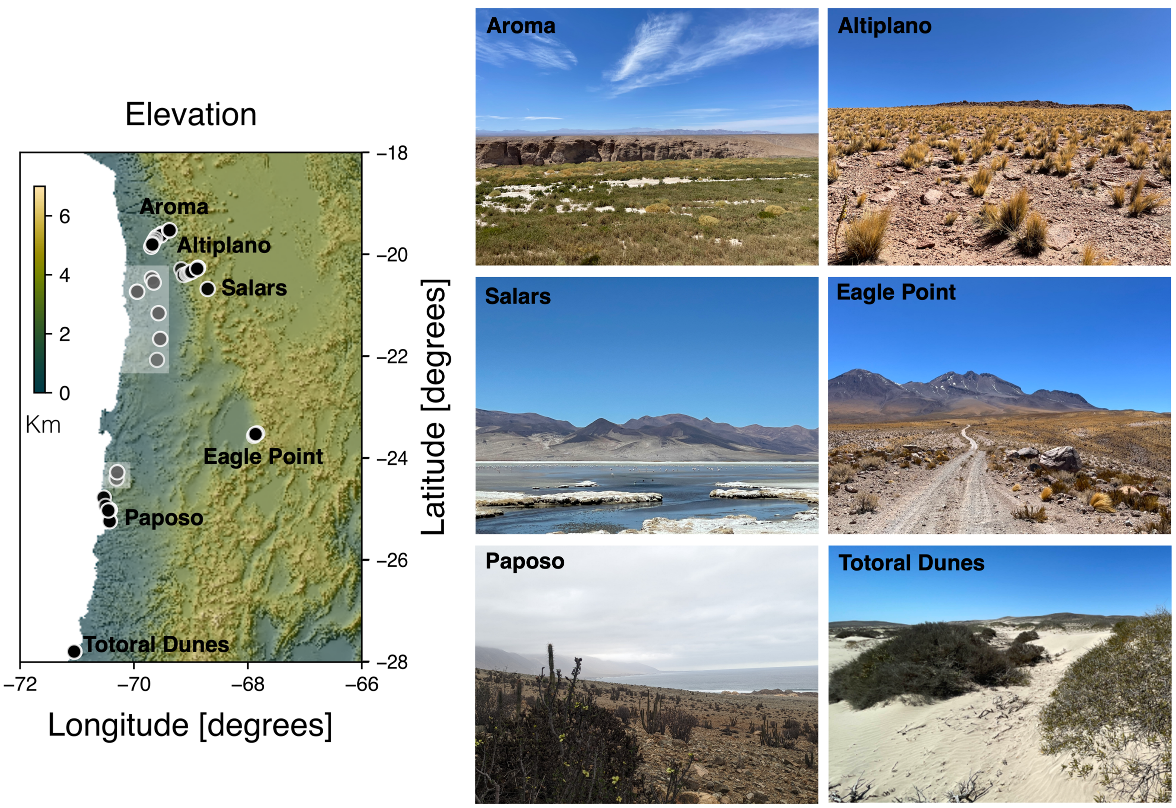

Fig. S1. Locations and sampling spots distributed across the Atacama Desert. Shading in the map corresponds to elevation in Km above sea level. Sampling spots in white shadow boxes represent no formal transects, and areas where no nematodes were found. Pictures for each of the transects highlight the differences in the ecosystems. The Aroma transect is located near the Quebrada de Aroma, a fresh water stream and vegetation coverage is high. The Altiplano transect is a high altitude transect with lower vegetation and water streams with seasonal rainfall. The Salars transect(including Salar de Huasco and Laguna Grande) correspond to wetlands with high salinity patches and fresh water inflow. The Eagle Point transect is a high altitude transect with lower vegetation. The Paposo transect is close the coast and receives moisture in the form of fog and has high plant endemism. The Totoral Dunes transect is also located close to the cost and is characterized by sandy dunes. Photographs of the desert where taken by the manuscript's authors.

Table S1. Sampling coordinates of Totoral Dunes (TDT) locality

Locality	Longitud	Latitude
TDT	-71,04472222	-27,79444444
TDT	-71,04472222	-27,79416667
TDT	-71,045	-27,79416667
TDT	-71,04527778	-27,79416667
TDT	-71,04555556	-27,79416667
TDT	-71,04611111	-27,79416667
TDT	-71,04638889	-27,79416667
TDT	-71,04694444	-27,79388889
TDT	-71,04722222	-27,79388889
TDT	-71,04777778	-27,79388889
TDT	-71,04805556	-27,79388889
TDT	-71,04833333	-27,79388889
TDT	-71,04888889	-27,79361111
TDT	-71,04916667	-27,79361111
TDT	-71,04944444	-27,79361111
TDT	-71,04944444	-27,79361111
TDT	-71,04972222	-27,79333333
TDT	-71,05027778	-27,79361111
TDT	-71,05027778	-27,79333333
TDT	-71,05055556	-27,79333333

Table S2. Sampling coordinates of Paposo (PAP) locality

Locality	Longitude	Latitude
PAP	-70,423056	-25,014167
PAP	-70,423056	-25,014167
PAP	-70,425833	-25,011667
PAP	-70,428333	-25,012778
PAP	-70,429444	-25,018333
PAP	-70,532222	-24,772778
PAP	-70,530833	-24,7725
PAP	-70,498056	-24,935
PAP	-70,425	-25,233889
PAP	-70,425278	-25,235
PAP	-70,426389	-25,235556
PAP	-70,427778	-25,236944
PAP	-70,445	-25,021389
PAP	-70,450833	-25,024444
PAP	-70,451111	-25,025

Table S3. Sampling coordinates of Eagle Point transect (EPT) locality

Locality	Longitude	Latitude
EPT	-67,892222	-23,563056
EPT	-67,877222	-23,541944
EPT	-67,860556	-23,521667
EPT	-67,848889	-23,516111
EPT	-67,834444	-23,510556
EPT	-67,865278	-23,526667
EPT	-67,865278	-23,526667
EPT	-67,865278	-23,526667
EPT	-67,865278	-23,526667
EPT	-67,865278	-23,526667
EPT	-67,865278	-23,526667
EPT	-67,865278	-23,526667
EPT	-67,865278	-23,526667
EPT	-67,865278	-23,526667
EPT	-67,865278	-23,526667
EPT	-67,865278	-23,526667
EPT	-67,865278	-23,526667
EPT	-67,865278	-23,526667
EPT	-67,865278	-23,526667
EPT	-67,865278	-23,526667
EPT	-67,865278	-23,526667

Table S4. Sampling coordinates of Aroma (ARO) locality

Locality	Longitude	Latitude
ARO	-69,677778	-19,807222
ARO	-69,677778	-19,807222
ARO	-69,677778	-19,807222
ARO	-69,608611	-19,665833
ARO	-69,591944	-19,654722
ARO	-69,581667	-19,644722
ARO	-69,507222	-19,595833
ARO	-69,510556	-19,595278
ARO	-69,510556	-19,595278
ARO	-69,381111	-19,528611
ARO	-69,381111	-19,528611
ARO	-69,3775	-19,53
ARO	-69,378611	-19,528056
ARO	-69,374167	-19,526667
ARO	-69,526111	-19,625556
ARO	-69,526111	-19,625556
ARO	-69,528889	-19,626389
ARO	-69,626111	-19,697778
ARO	-69,648889	-19,739167
ARO	-69,667222	-19,781111
ARO	-69,695	-19,849722
ARO	-69,695	-19,849722
ARO	-69,696389	-19,849722

Table S5. Sampling coordinates of Altiplano (ALT) locality

Locality	Longitude	Latitude
ALT	-69,126667	-20,405278
ALT	-69,069722	-20,381389
ALT	-69,042778	-20,369167
ALT	-69,010556	-20,355
ALT	-68,975278	-20,339722
ALT	-68,991944	-20,339722
ALT	-69,168333	-20,291667

Table S6. Sampling coordinates of the Salars locality

Locality	Longitude	Latitude
Salars	-68,875556	-20,2625
Salars	-68,875556	-20,2625
Salars	-68,886984	-20,276002
Salars	-68,886959	-20,275679
Salars	-68,875961	-20,26247
Salars	-68,875839	-20,262499
Salars	-68,889651	-20,282766
Salars	-68,889289	-20,28312
Salars	-68,889919	-20,282545
Salars	-68,706111	-20,678333
Salars	-68,705833	-20,678611

Table S7. Sampling coordinates in areas outside the defined sampling localities.

Locality	Longitude	Latitude
TAM	-69,686389	-20,472778
TAM	-69,686389	-20,472778
TAM	-69,653333	-20,553611
TAM	-69,653611	-20,553056
RL	-69,535556	-21,658056
RL	-69,540278	-21,66
MET	-69,595833	-22,070556
CDM	-70,304444	-24,398611
CDM	-70,293333	-24,275556
CDM	-70,293333	-24,275556
BTM	-69,568333	-21,154167
BTM	-69,568333	-21,154167
acCPAJ	-69,945556	-20,732778
acCPAJ	-69,945278	-20,7325
acCPAJ	-69,941667	-20,7325

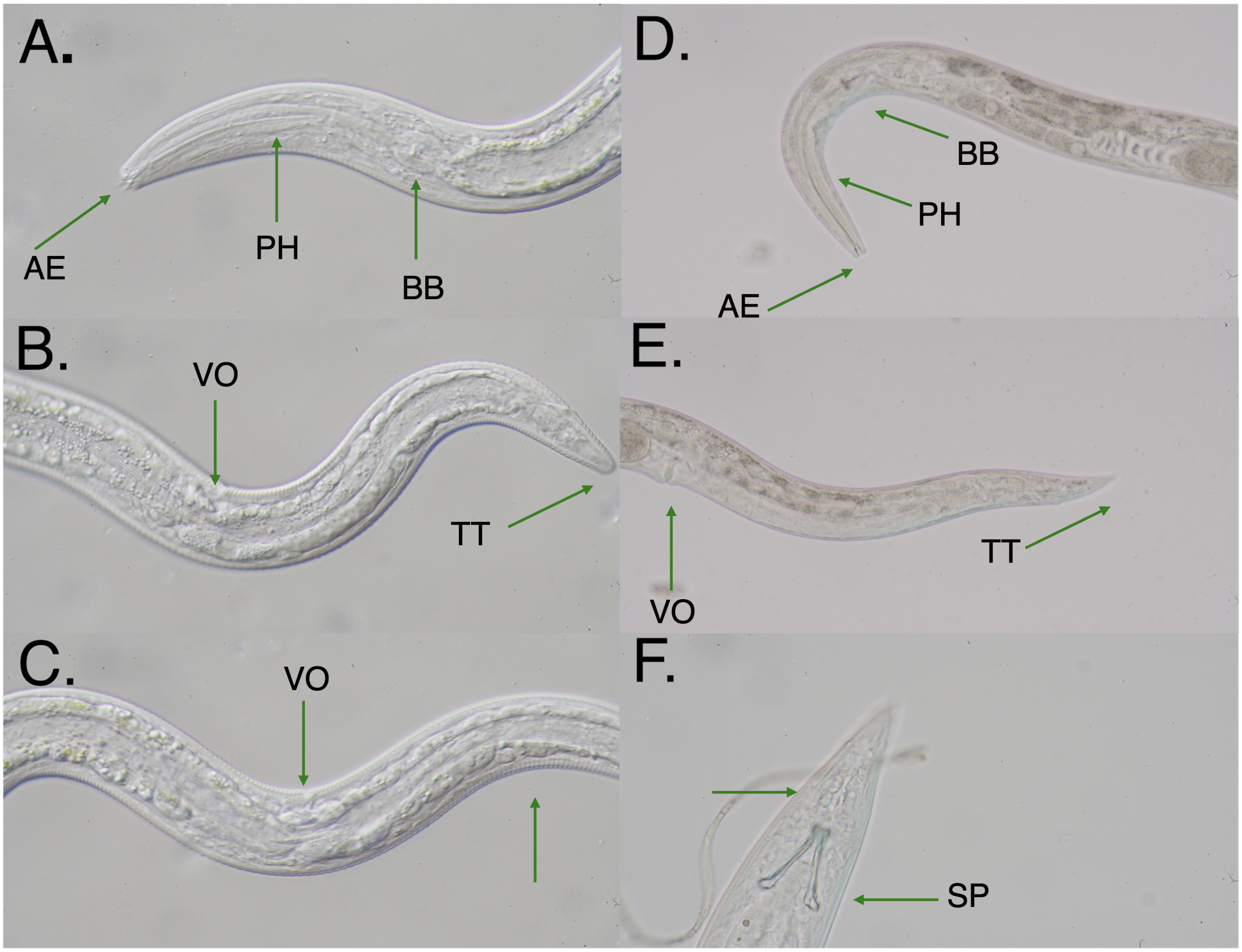

Fig. S2. Morphological characters used for the identification of extracted nematodes to families and genera. Exemplified by *Acrobeloides* (A-C) and *Panagrolaimus* (D-F). In panel A, a complex labial region can be seen (AE), and in E a simple labial region . *Diagnostic features: shape and size of stoma, presence or absence of tooth or odontostyle and labial structure.* In A and D both cases the presence of a well developed muscular Pharynx (PH) followed by a basal bulb with a grinder (BB). *Diagnostic features: musculature, shape of the pharynx and presence or absence and structure of bulb.* In B and E the vulva opening is indicated (VO) in female individuals in both cases, the tail tip differs (TT) between B and F, round and conoid tail, respectively. *Diagnostic features: reproductive structures and its position relative to whole body length, number of gonads, shape and length of tail.* In F a male individual is seen identified by the presence of a spicule (SP), in contrast the identified species in A-C only displayed females individuals as seen from morphological structures (VO) and experimental work, additionally in C a coarsely annulated cuticle is seen and in F a finely annulated cuticle. *Diagnostic features: presence and shape of spicule and cuticle structure*

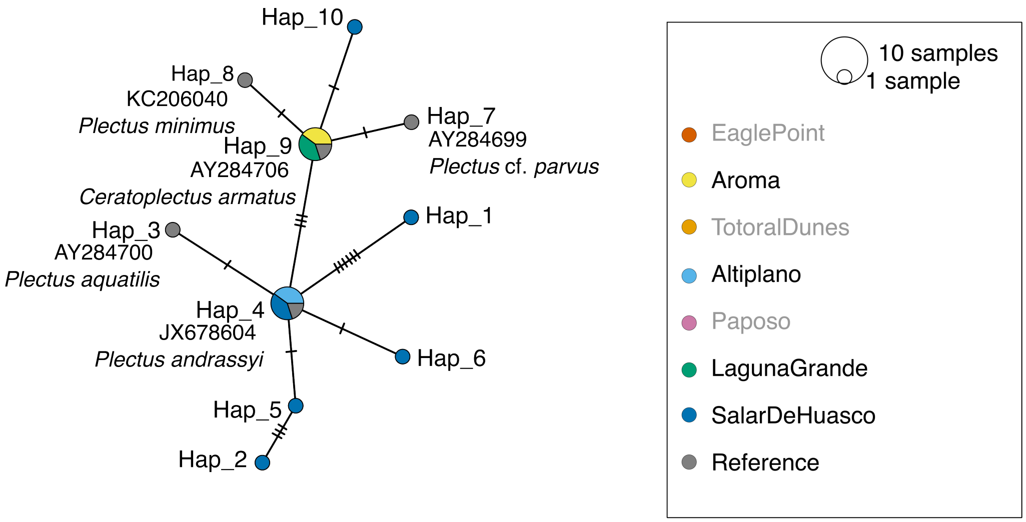

Fig. S3. Haplotype network for Plectidae. Based on 18S rRNA sequences. Color refers to sampling locations. Locations in grey are not present. Reference sequences were retrieved from the curated 18S-NemaBase (1). Accession number and species name is written next to the respective node of haplotype (abbreviated to "Hap"). Vertical hatchmarks indicate the number of differences from one node to the next.

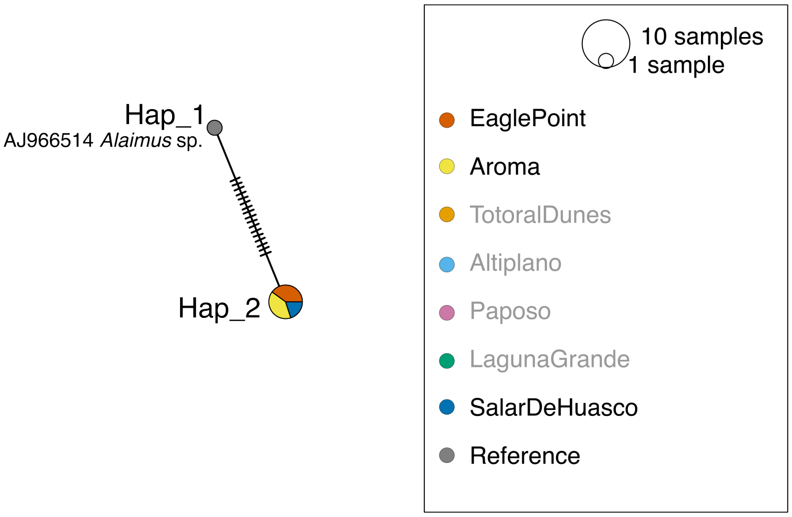

Fig. S4. Haplotype network for Alaimidae. Based on 18S rRNA sequences. Color refers to sampling locations. Locations in grey are not present. Reference sequences were retrieved from the curated 18S-NemaBase (1). Accession number and species name is written next to the respective node of haplotype (abbreviated to "Hap"). Vertical hatchmarks indicate the number of differences from one node to the next.

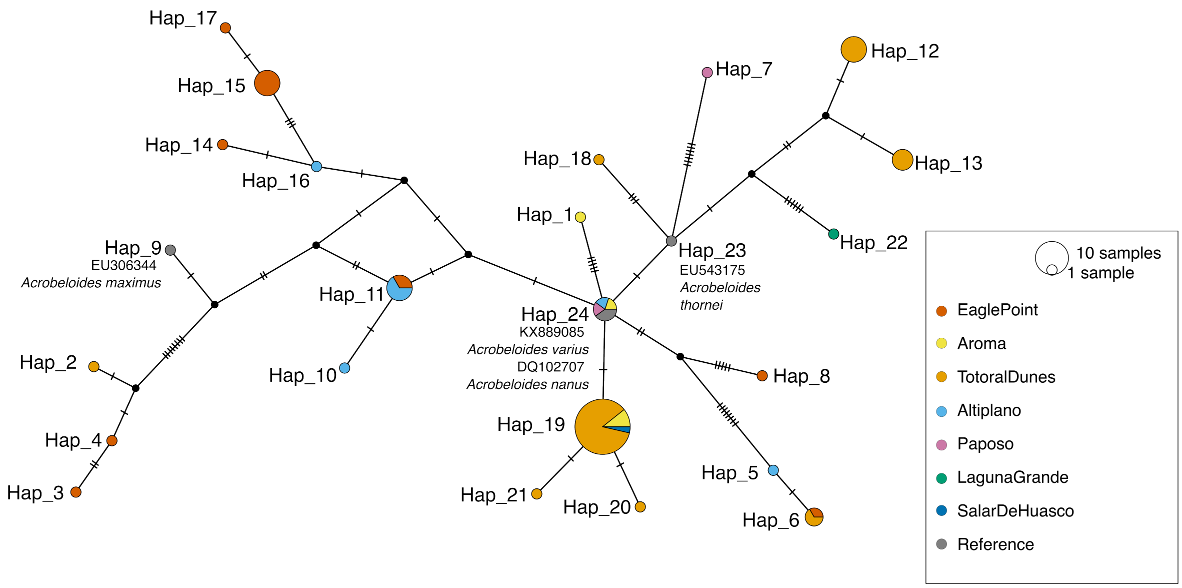

Fig. S5. Haplotype network for *Acrobeloides*. Based on 18S rRNA sequences. Color refers to sampling locations. Reference sequences were retrieved from the curated 18S-NemaBase (1). Accession number and species name is written next to the respective node of haplotype (abbreviated to "Hap"). Vertical hatchmarks indicate the number of differences from one node to the next.

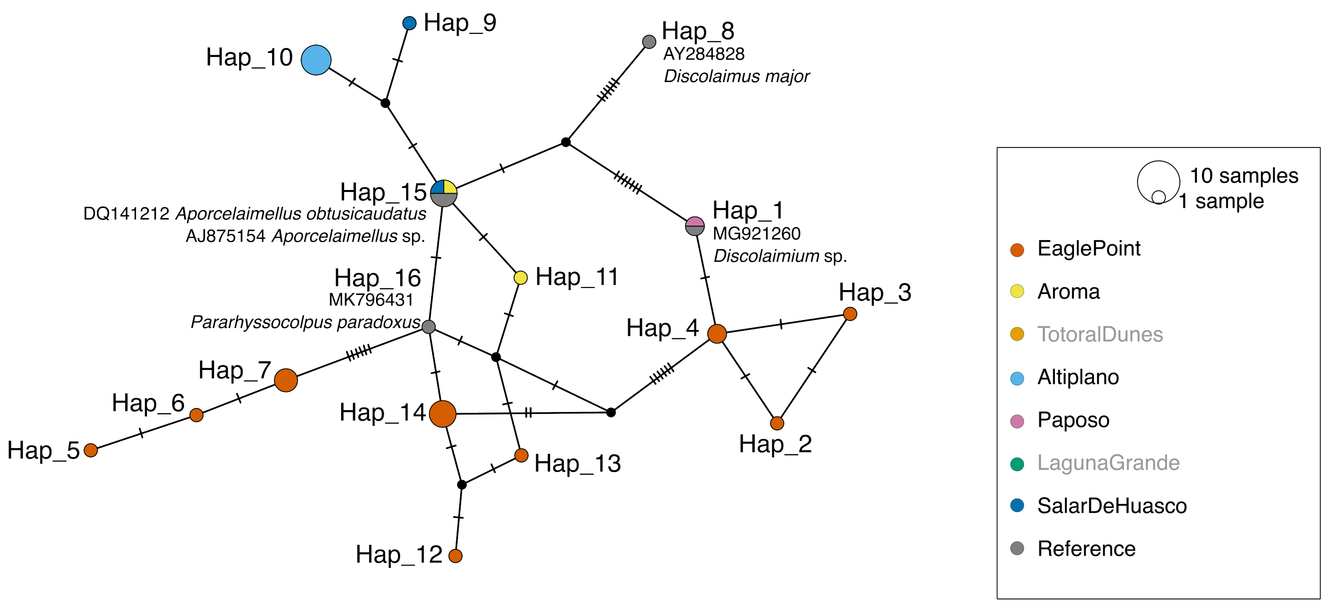

Fig. S6. Haplotype network for Dorylamida. Based on 18S rRNA sequences. Color refers to sampling locations. Locations in grey are not present. Reference sequences were retrieved from the curated 18S-NemaBase (1). Accession number and species name is written next to the respective node of haplotype (abbreviated to "Hap"). Vertical hatchmarks indicate the number of differences from one node to the next.

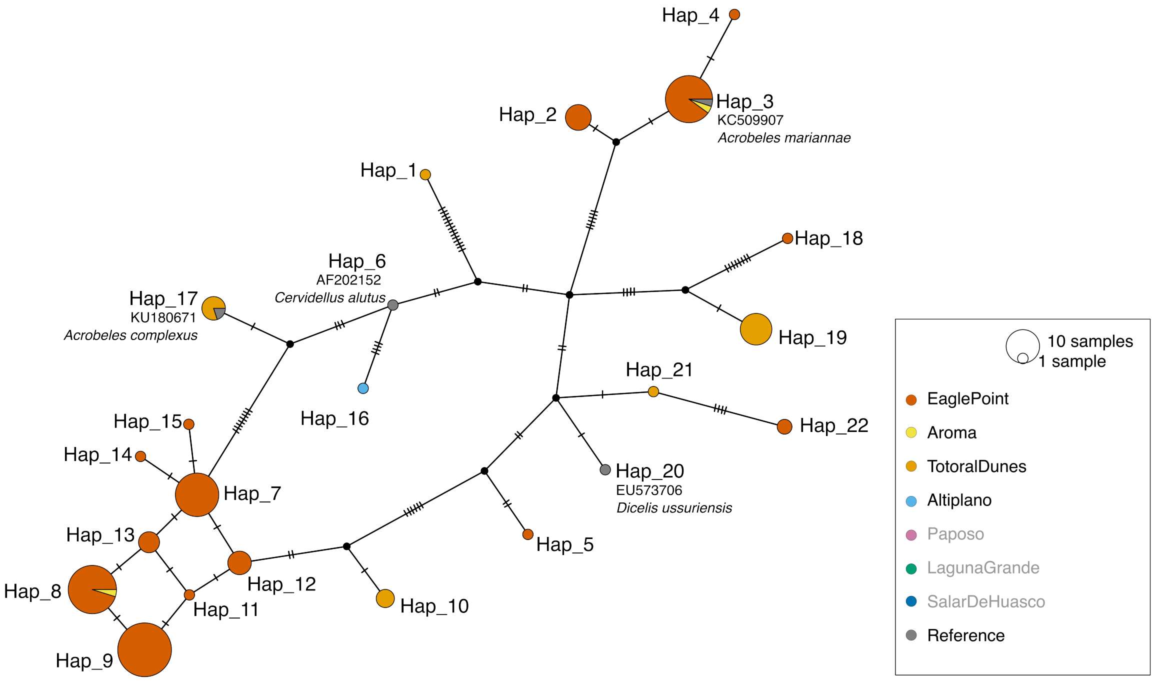

Fig. S7. Haplotype network for *Acrobeles*. Based on 18S rRNA sequences. Color refers to sampling locations. Locations in grey are not present. Reference sequences were retrieved from the curated 18S-NemaBase (1). Accession number and species name is written next to the respective node of haplotype (abbreviated to "Hap"). Vertical hatchmarks indicate the number of differences from one node to the next.

Table S8. Details on the curated alignments used for haplotype networks, total nucleotide diversity (π) and total genetic diversity (Watterson's estimator, θ).

Taxonomic group	Number of sequences	Alignment length (bp)	Number of segregating sites
Plectidae	18	533	18
Alaimidae	6	497	17
Acrobeloides	75	487	51
Dorylamida	30	421	24
Acrobeles	129	328	47
Panagrolaimus	47	367	67
Aphelenchoidea	20	645	166

Table S9. Details on nucleotide diversity (π) and genetic diversity (Watterson's estimator, θ) for each taxonomic group based on every location. The number of sequences and number of haplotypes per location are also specified. The geographic distance of the different regions was tested against the genetic distance (Euclidean distance) with Mantel statistics based on Spearman's rank correlation (ρ). The references were excluded from the statiscal analyses (grey column). Significant p-values (p <0.05) are marked with an asterix.

Taxonomic group	Measurement	Eagle Point	Aroma	Totoral Dunes	Altiplano	Paposo	Salars	Reference	Mantel statistics
Plectidae	π	NA	0	NA	0	NA	0.0082	0.00568	r = 0, p = 0.66667, 5 permutations
	θ	NA	0	NA	0	NA	0.0104	0.00545	
	Haplotypes	NA	1	NA	1	NA	7	5	
	Sequences	0	2	0	2	0	9	5	
Alaimidae	π	0	0	NA	NA	NA	NA	NA	NA
	θ	0	0	NA	NA	NA	NA	NA	
	Haplotypes	1	1	NA	NA	NA	1	1	
	Sequences	2	2	0	0	0	1	1	
Acrobeloides	π	0.01938	0.0063	0.00932	0.00959	0.0186	0.0186	0.00723	r = -0.07527, p = 0.55278, 719 permutations
	θ	0.01935	0.007	0.01457	0.01195	0.0186	0.0186	0.00789	
	Haplotypes	8	3	8	5	2	2	3	
	Sequences	14	5	40	8	2	2	4	
Dorylamida	π	0.01788	0.0024	NA	0	NA	0.0048	0.01449	r = 0.7143, p = 0.16667, 23 permutations
	θ	0.0112	0.0024	NA	0	NA	0.0048	0.01596	
	Haplotypes	9	2	NA	1	1	2	4	
	Sequences	15	2	0	5	1	2	5	
Acrobeles	π	0.02342	0.0491	0.03136	NA	NA	NA	0.0317	r = -0.5, p = 0.83333, 5 permutations
	θ	0.0194	0.0491	0.02943	NA	NA	NA	0.03012	
	Haplotypes	14	2	5	1	NA	NA	4	
	Sequences	104	2	18	1	0	0	4	
Panagrolaimus	π	0.06548	0.055	0	0.05476	0.03217	NA	0.04137	r = 0.6, p = 0.041667 *, 119 permutations
	θ	0.06144	0.055	0	0.04056	0.02913	NA	0.04603	
	Haplotypes	3	3	1	8	5	NA	4	
	Sequences	4	3	5	15	13	0	5	
Aphelenchoidea	π	0.05241	0.0634	NA	NA	NA	NA	0.1135	NA
	θ	0.04538	0.0836	NA	NA	NA	NA	0.09555	
	Haplotypes	5	5	NA	1	1	NA	6	
	Sequences	6	6	0	1	1	0	6	

Table S10. Summary of barcoding data (18S rRNA). Sorted by locality and assigned group for TCS haplotype networks. Number of haplotype per group and locality is given, supported by the assigned haplotype number of the networks (Hap). Molecular based identification according to best hit (lowest E-value and a high percentage of identity) against the curated 18S-NemaBase is specified. Localities are abbreviated as follow: Eagle Point as "EPT", Aroma as "ARO", Totoral Dunes as "TDT", Altiplano as "ALT", Paposo as "PAP", Salars are split into two sublocations "LAG" for Laguna Grande and "SLH" for Salar de Husaco.

Locality	Haplotype ID	Count Haplotypes	Haplotypes (Hap)	Molecular Identification	Count Families	Families
**EPT**	*Acrobeles*-like	14	2, 3, 4, 5, 7, 8, 9, 11, 12, 13, 14, 15, 18, 22	*Acrobeles* sp., *Acrobeloides* sp.	1	Cephalobidae
	Acrobeloides	8	3, 4, 6, 8, 11, 14, 15, 17	*Acrobeloides* sp.	1	Cephalobidae
	Alaimidae	1	2	*Alaimus* sp.	1	Alaimidae
	Aphelenchoidea	4	1, 6, 7, 10	Aphelenchoididae sp., *Aphelenchoides* sp.	1	Aphelenchoididae
	Dorylaimida	9	2, 3, 4, 5, 6, 7, 12, 13, 14	Dorylaimida sp., Dorylaimina sp., Discolaimium sp.	4	Aporcelaimidae, Pararhyssocolpidae, Qudsianematidae, Undefined
	Panagrolaimus	3	5, 7, 19	*Panagrolaimus* sp.	1	Panagrolaimidae
	**total**	**39**			**9**	
**ARO**	*Acrobeles*-like	2	3, 8	*Acrobeles* sp.	1	Cephalobidae
	Acrobeloides	3	1, 19, 24	*Acrobeloides* sp.	1	Cephalobidae
	Alaimidae	1	2	*Alaimus* sp.	1	Alaimidae
	Aphelenchoidea	5	2, 13, 14, 15, 16	*Aphelenchoides* sp.	1	Aphelenchoididae
	Dorylaimida	2	11, 15	Dorylaimida sp.	1	Aporcelaimidae
	Panagrolaimus	3	5, 19, 20	*Panagrolaimus* sp.	1	Panagrolaimidae
	Plectidae	1	9	*Plectus* sp.	1	Plectidae
	**total**	**17**			**7**	
**TDT**	*Acrobeles*-like	5	1, 10, 17, 19, 21	*Acrobeles* sp.	1	Cephalobidae
	Acrobeloides	8	2, 6, 12, 13, 18, 19, 20, 21	*Acrobeloides* sp.	1	Cephalobidae
	Panagrolaimus	1	16	*Panagrolaimus* sp.	1	Panagrolaimidae
	**total**	**14**			**3**	
**ALT**	*Acrobeles*-like	1	16	*Acrobeles* sp.	1	Cephalobidae
	Acrobeloides	5	5, 10, 11, 16, 24	*Acrobeloides* sp.	1	Cephalobidae
	Aphelenchoidea	1	10	*Aphelenchoides* sp.	1	Aphelenchoididae
	Dorylaimida	1	10	Dorylaimida sp.	1	Aporcelaimidae
	Panagrolaimus	8	2, 5, 6, 11, 12, 13, 14, 15	*Panagrolaimus* sp.	1	Panagrolaimidae
	Plectidae	1	4	*Plectus* sp.	1	Plectidae
	**total**	**17**			**6**	
**PAP**	*Acrobeloides*	2	7, 24	*Acrobeloides* sp.	1	Cephalobidae
	Aphelenchoidea	1	5	*Aphelenchoides* sp.	1	Aphelenchoididae
	Dorylaimida	1	1	Dorylaimida sp.	1	Aporcelaimidae
	Panagrolaimus	5	1, 4, 16, 17, 18	*Panagrolaimus* sp.	1	Panagrolaimidae
	**total**	**9**			**4**	
**LAG**	*Acrobeloides*	1	22	*Acrobeloides* sp.	1	Cephalobidae
	Plectidae	1	9	*Plectus* sp.	1	Plectidae
	**total**	**2**			**2**	
**SLH**	*Acrobeloides*	1	19	*Acrobeloides* sp.	1	Cephalobidae
	Alaimidae	1	2	*Alaimus* sp.	1	Alaimidae
	Dorylaimida	2	9, 15	Dorylaimida sp.	2	Aporcelaimidae, Dorylaimidae
	Plectidae	6	1, 2, 4, 5, 6, 10	*Plectus* sp.	1	Plectidae
	**total**	**10**			**5**	
**NCBI References**	*Acrobeles*-like	4	3, 6, 17, 20		2	Cephalobidae, Drilonematidae
	Acrobeloides	3	9, 23, 24		1	Cephalobidae
	Alaimidae	1	1		1	Cephalobidae
	Aphelenchoidea	6	3, 4, 8, 9, 11, 12		1	Aphelenchoididae, Aphelenchidae
	Dorylaimida	4	1, 8, 15, 16		3	Qudsianematidae, Aporcelaimidae, Pararhyssocolpidae
	Panagrolaimus	4	3, 8, 9, 10		1	Panagrolaimidae
	Plectidae	5	3, 4, 7, 8, 9		1	Plectidae

Table S11. Colonizer persister category (CP) proportion of the families found in different sampling transects.

	**Altiplano**	**Aroma**	**EaglePoint**	**Paposo**	**Salars**	**TotoralDunes**
CP 1	14.3	14.3	12.5	11.1	9.1	28.6
CP 2	42.9	42.9	37.5	33.3	27.3	28.6
CP 3	0.0	0.0	0.0	0.0	18.2	0.0
CP 4	28.6	28.6	37.5	33.3	36.4	28.6
CP 5	14.3	14.3	12.5	22.2	9.1	14.3
CP1+CP2	*57.2*	*57.2*	*50*	*44.4*	*36.4*	*57.2*
CP4+CP5	*42.9*	*42.9*	*50*	*55.5*	*45.5*	*42.9*

Table S12. Feeding types of the families found in the different sampling transects.

	Altiplano	Aroma	Eagle Point	Paposo	Salars	Totoral Dunes
Herbivores	0.0	0.0	11.1	10.0	0.0	12.5
Fungivores	28.6	14.3	11.1	20.0	0.0	12.5
Bacterivores	42.9	57.1	44.4	20.0	45.5	37.5
Predators	14.3	14.3	11.1	20.0	27.3	25.0
Unicelular eukaryotic feeders	0.0	0.0	0.0	0.0	0.0	0.0
Omnivores	14.3	14.3	22.2	30.0	27.3	12.5

Table S13. Geomorphological and climatic variables tested for the modeling approaches.

Variable	Description	Type	Kind	min	max
Response	Composition of genera	integer	Biotic	0	6
Predictive	Elevation	numeric	Geomorphological	84	4341
Predictive	Topographic complexity	numeric	Geomorphological	1,52819	214,6917
Predictive	Soil thickness	numeric	Geomorphological	0	50
Predictive	Curvature	numeric	Geomorphological	-3200	900
Predictive	Rock type	integer	Geomorphological	1	11
Predictive	Latitude	numeric	Geomorphological	-27.79444	-19.52667
Predictive	Mean annual temperature	numeric	Climatic	4.325012	21.75835
Predictive	Range of temperature	numeric	Climatic	2.700012	6.799988
Predictive	Mean annual precipation	numeric	Climatic	3.3	311.2
Predictive	Range of precipitation	numeric	Climatic	0.7	91.799995

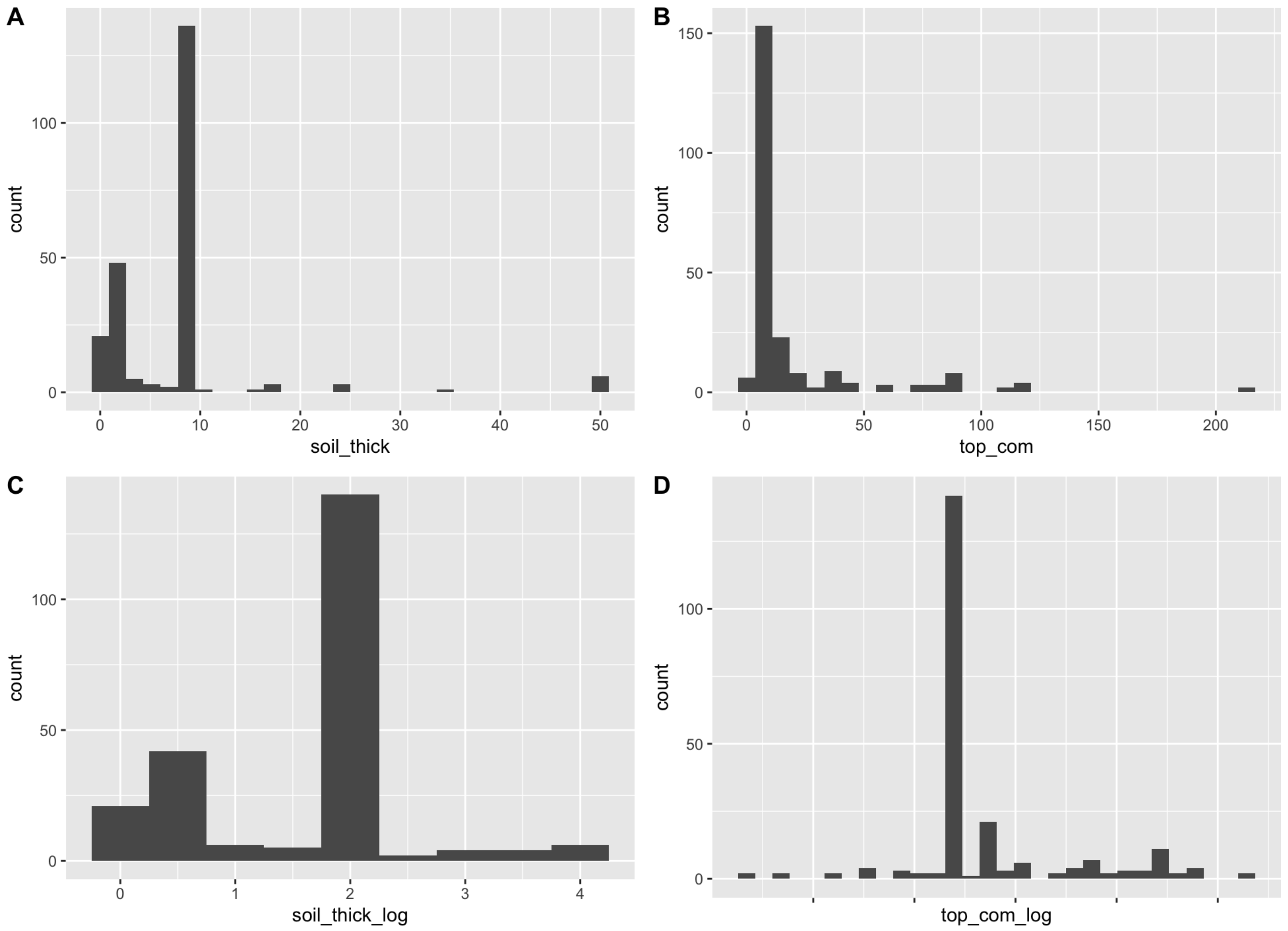

Fig. S8. Variable transformation for modeling. Transformation of soil thickness (A and C) and topographic complexity (B and D) for implementation in generalised linear model.

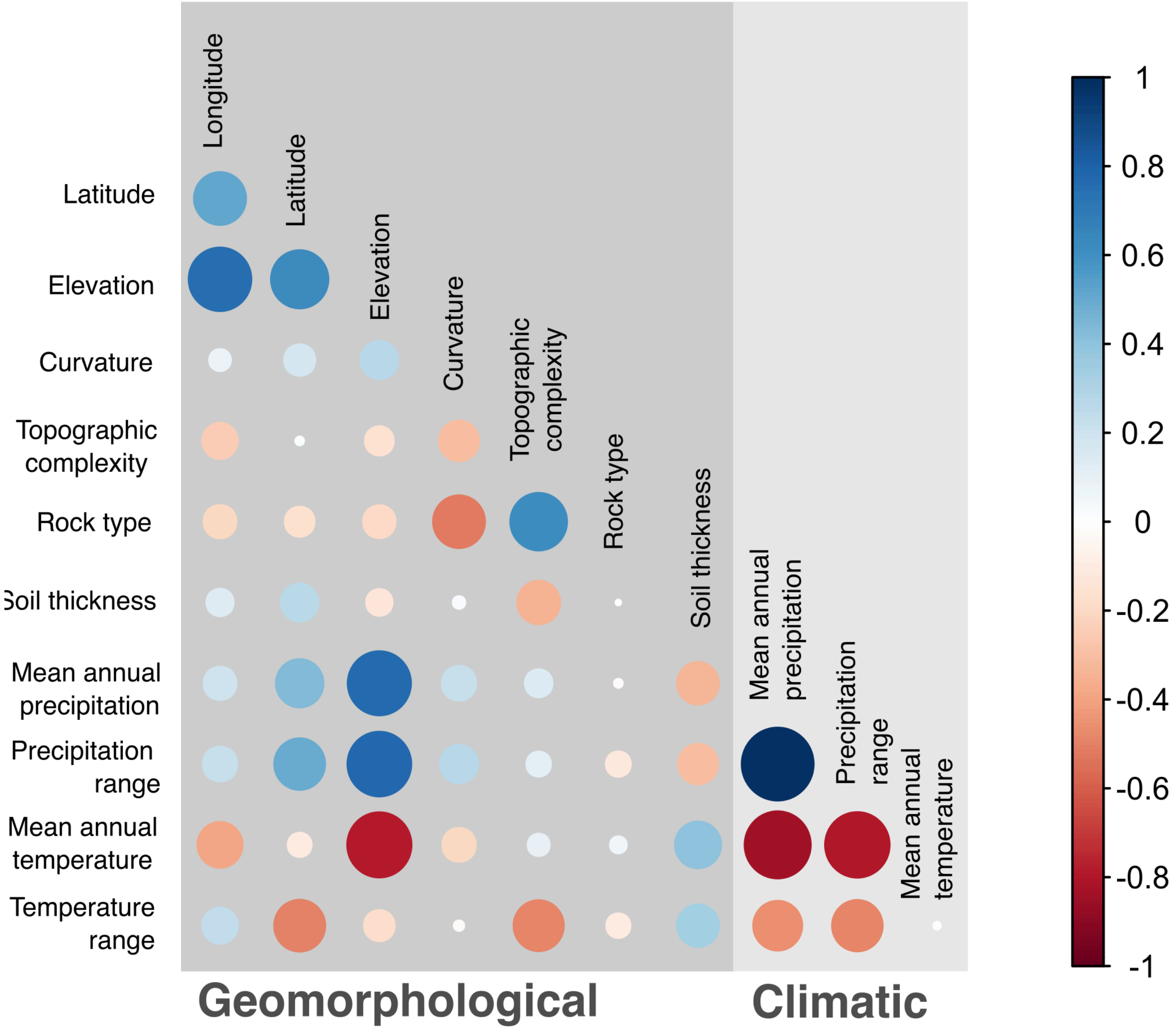

Fig. S9. Possible predictors of genera richness. Correlation of geographical and climatic variables considered as possible predictors in the Generalized Least Squares models(GLS), Linear mixed-effects models (LME) and Random forest approach to determine genera richness in the desert (Pearson method). Variables mean annual precipitation and precipitation range show a high positive correlation (0.9884, $p<0.05$). Similarly, mean annual temperature and mean annual precipitation exhibit a strong negative correlation (-0.8357, $p<0.05$). Additionally, mean annual temperature and precipitation range also display a high negative correlation (-0.7967, $p<0.05$). Based on these correlations, the variables selected for further analysis include latitude, elevation, curvature, topographic complexity, rock type, soil thickness, mean annual precipitation, and range of temperature. High negative correlation is depicted in dark red and high positive correlation is depicted in dark blue.

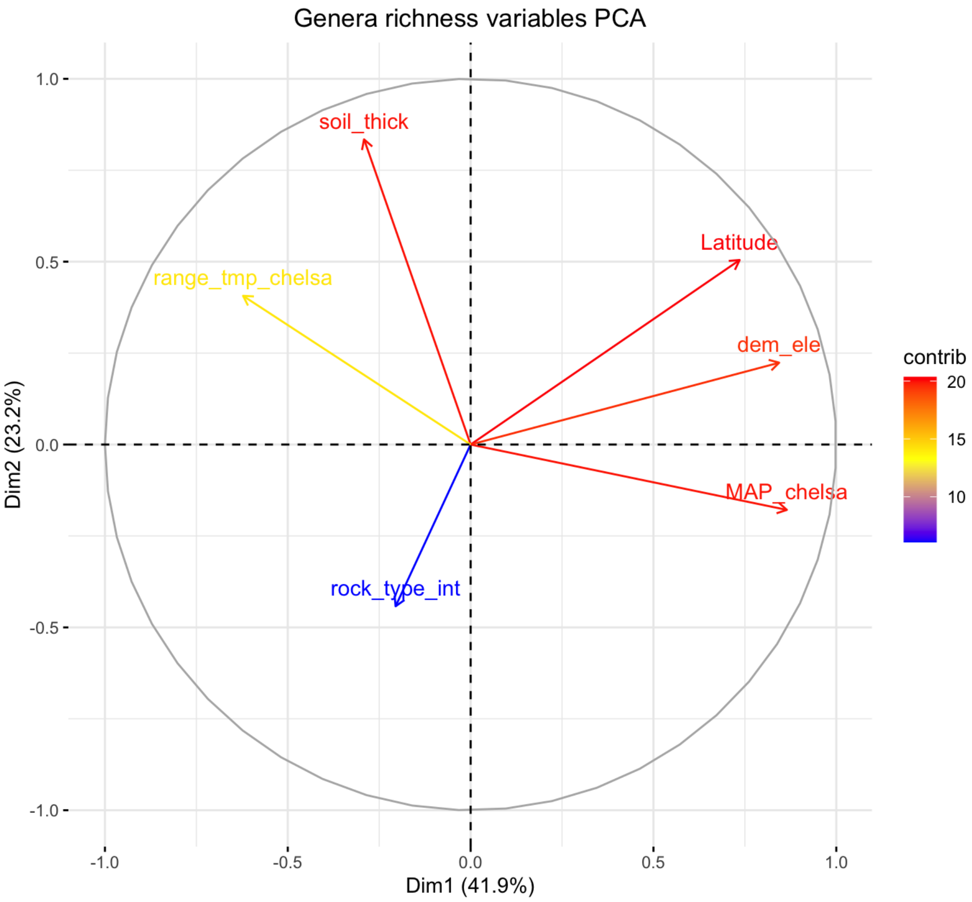

Fig. S10. Principal component analysis of variables to be used as predictor for genera richness in general linear models. Variable contribution ("contrib") is low when depicted in blue, and high when depicted in red.

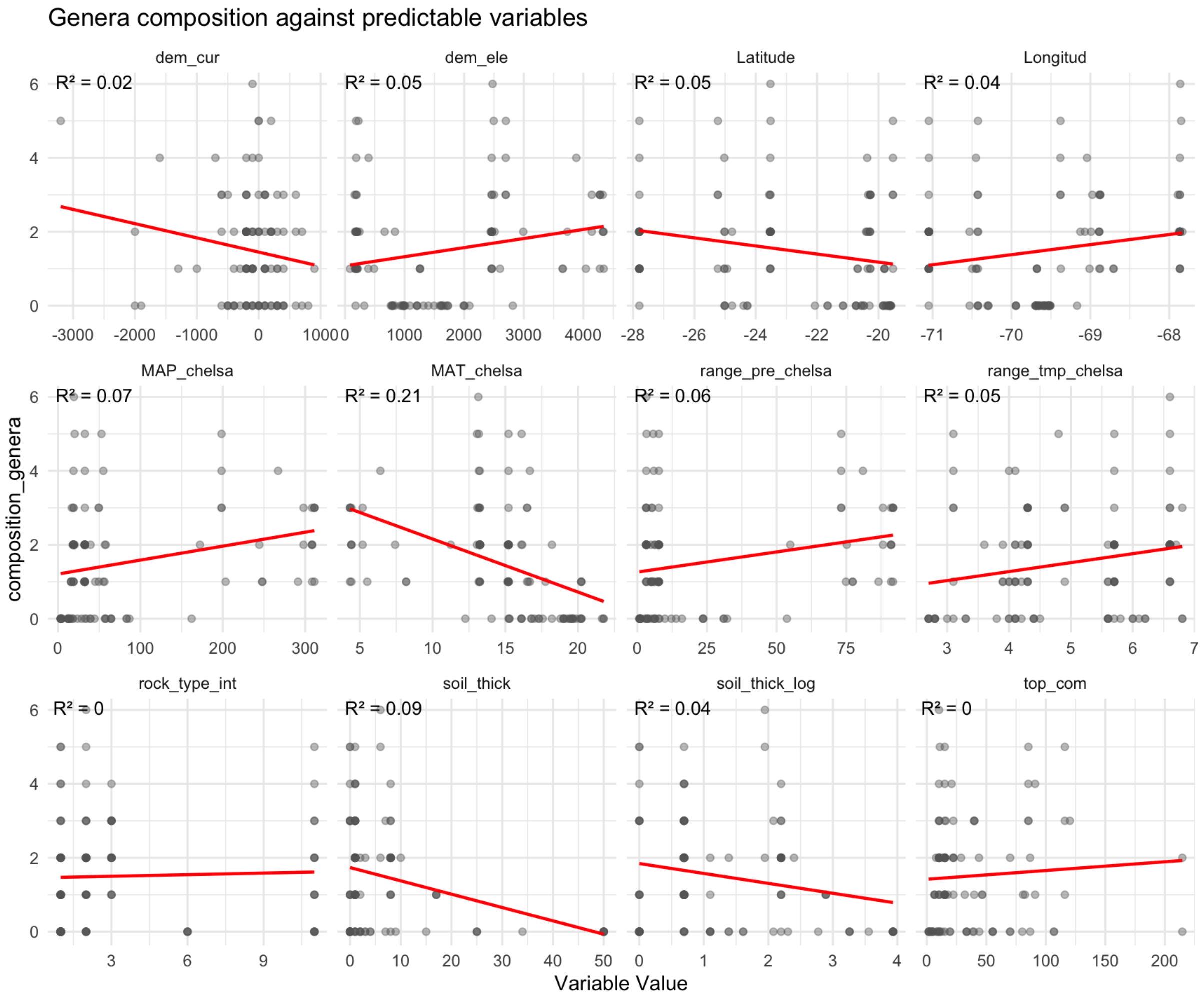

Fig. S11. Scatter plots showing the relationships between environmental variables and nematode genus richness across sampling sites in the Atacama Desert. Each panel displays a fitted linear regression line (in red) along with the corresponding R^2 value. While most relationships appear weak or non-linear, these plots illustrate the direct (bivariate) associations in the raw data and emphasize the limitations of linear assumptions.

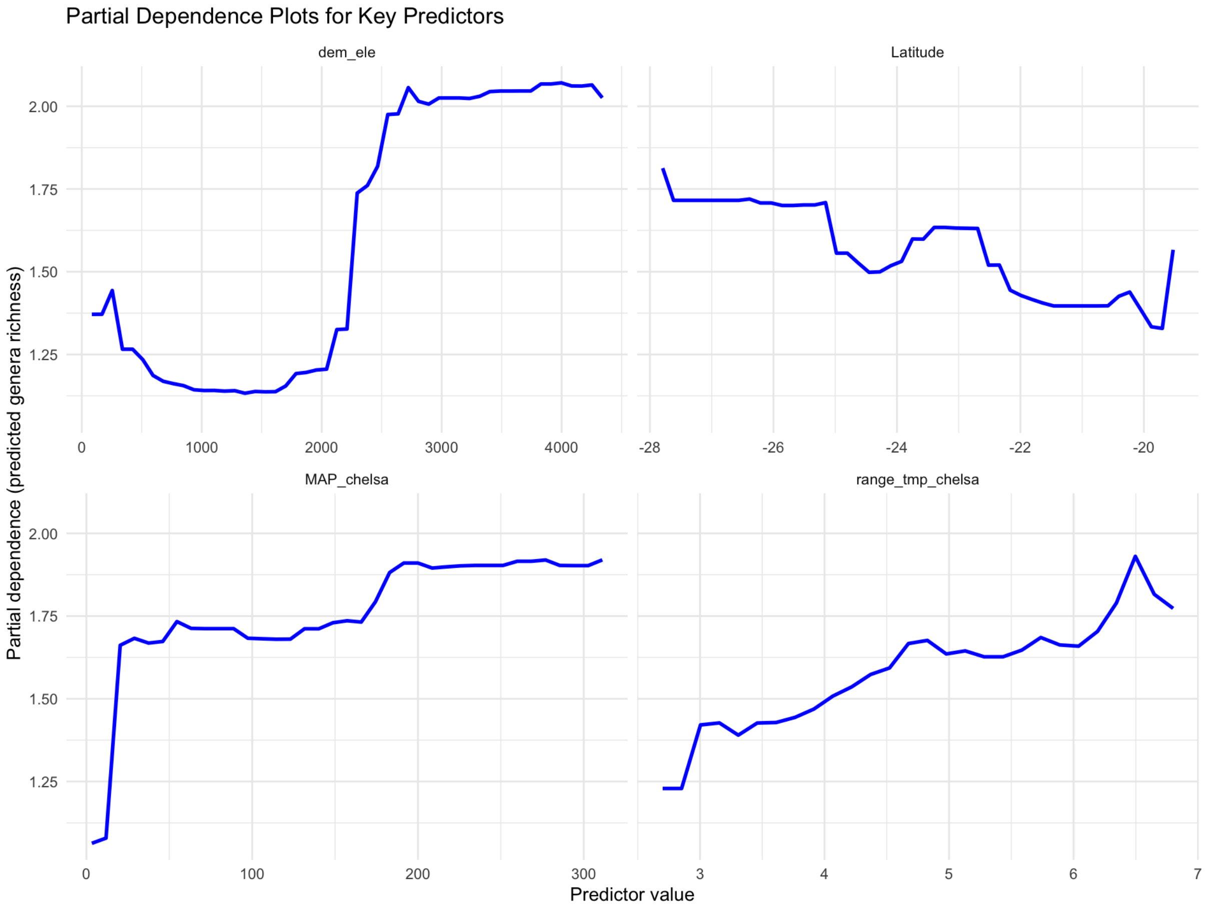

Fig. S12. Partial dependence plots showing how predicted nematode genus richness responds to changes in individual environmental variables, based on a random forest model. Each plot illustrates the effect of one predictor while averaging over the others. The nonlinear shapes reveal complex model responses, highlighting patterns not fully captured by linear models.

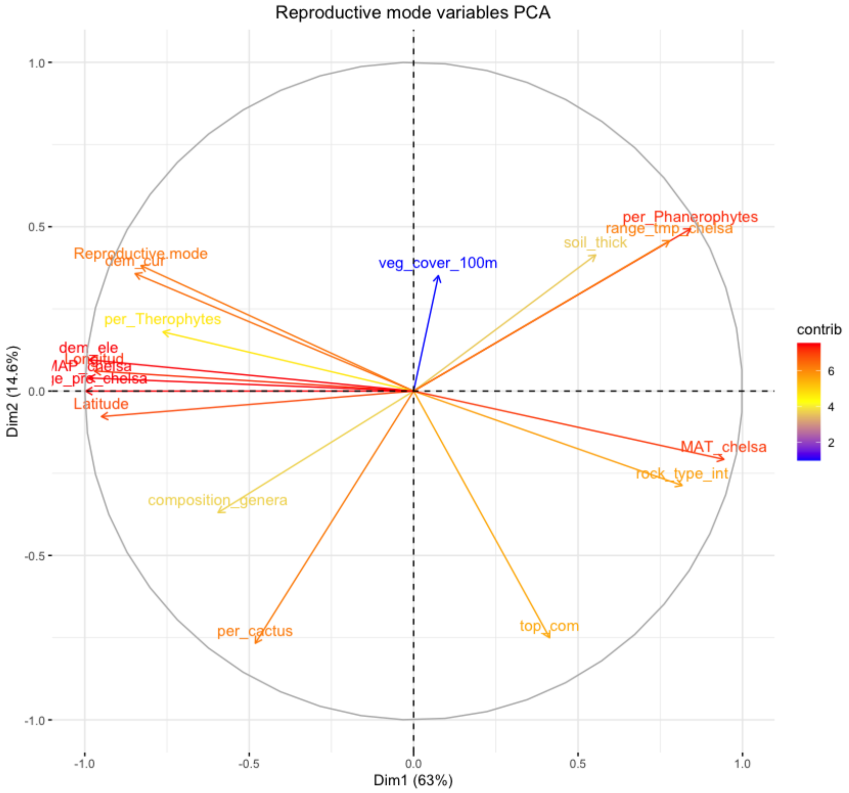

Fig. S13. Principal component analysis of variables to be used as predictor for reproductive mode in general linear models. Variable contribution ("contrib") is low when depicted in blue, and high when depicted in red.

Table S14. Generalized least squares model specifications for genera richness displaying combination of variables tested. The best model (M_4_14), incorporation latitude and mean annual precipitation) is highlighted and was chosen according to the lowest BIC and highest akaike weight.

Model	Model specification using GLS	BICs	Akaike weights
M_1_0	Latitude + dem_ele + rock_type_int + soil_thick_log + MAP_chelsa + range_tmp_chelsa	439,3296	2,50E-03
M_1_1	Latitude + dem_ele + rock_type_int + soil_thick_log + MAP_chelsa	435,7193	1,52E-02
M_1_2	Latitude + dem_ele + rock_type_int + soil_thick_log + range_tmp_chelsa	426,1046	1,86E+00
M_1_3	Latitude + dem_ele + rock_type_int + MAP_chelsa + range_tmp_chelsa	433,7931	3,98E-02
M_1_4	Latitude + dem_ele + soil_thick_log + MAP_chelsa + range_tmp_chelsa	425,7154	2,26E+00
M_1_5	Latitude + rock_type_int + soil_thick_log + MAP_chelsa + range_tmp_chelsa	422,8971	9,24E+00
M_1_6	dem_ele + rock_type_int + soil_thick_log + MAP_chelsa + range_tmp_chelsa	436,6535	9,52E-03
M_2_1	Latitude + dem_ele + rock_type_int + soil_thick_log	416,7643	1,98E+02
M_2_10	Latitude + soil_thick_log + MAP_chelsa + range_tmp_chelsa	409,2474	8,51E+03
M_2_11	dem_ele + rock_type_int + soil_thick_log + MAP_chelsa	452,9547	2,75E-06
M_2_12	dem_ele + rock_type_int + soil_thick_log + range_tmp_chelsa	424,355	4,46E+00
M_2_13	dem_ele + rock_type_int + MAP_chelsa + range_tmp_chelsa	437,3316	6,78E-03
M_2_14	dem_ele + soil_thick_log + MAP_chelsa + range_tmp_chelsa	427,9819	7,27E-01
M_2_15	rock_type_int + soil_thick_log + MAP_chelsa + range_tmp_chelsa	416,6406	2,11E+02
M_2_2	Latitude + dem_ele + rock_type_int + MAP_chelsa	429,0705	4,22E-01
M_2_3	Latitude + dem_ele + rock_type_int + range_tmp_chelsa	421,6383	1,73E+01
M_2_4	Latitude + dem_ele + soil_thick_log + MAP_chelsa	426,5662	1,48E+00
M_2_5	Latitude + dem_ele + soil_thick_log + range_tmp_chelsa	417,274	1,54E+02
M_2_6	Latitude + dem_ele + MAP_chelsa + range_tmp_chelsa	424,88	3,43E+00
M_2_7	Latitude + rock_type_int + soil_thick_log + MAP_chelsa	425,2025	2,92E+00
M_2_8	Latitude + rock_type_int + soil_thick_log + range_tmp_chelsa	422,7985	9,71E+00
M_2_9	Latitude + rock_type_int + MAP_chelsa + range_tmp_chelsa	413,251	1,15E+03
M_3_1	Latitude + dem_ele + rock_type_int	414,6345	5,75E+02
M_3_10	Latitude + MAP_chelsa + range_tmp_chelsa	403,9415	1,21E+05
M_3_11	dem_ele + rock_type_int + soil_thick_log	438,3542	4,07E-03
M_3_12	dem_ele + rock_type_int + MAP_chelsa	448,9546	2,03E-05
M_3_13	dem_ele + rock_type_int + range_tmp_chelsa	432,5371	7,45E-02
M_3_14	dem_ele + soil_thick_log + MAP_chelsa	444,0869	2,31E-04
M_3_15	dem_ele + soil_thick_log + range_tmp_chelsa	411,4559	2,82E+03
M_3_16	dem_ele + MAP_chelsa + range_tmp_chelsa	428,6293	5,26E-01
M_3_17	rock_type_int + soil_thick_log + MAP_chelsa	430,1279	2,49E-01
M_3_18	rock_type_int + soil_thick_log + range_tmp_chelsa	410,4978	4,55E+03
M_3_19	rock_type_int + MAP_chelsa + range_tmp_chelsa	412,6911	1,52E+03
M_3_2	Latitude + dem_ele + soil_thick_log	407,6717	1,87E+04
M_3_20	soil_thick_log + MAP_chelsa + range_tmp_chelsa	403,3468	1,63E+05
M_3_3	Latitude + dem_ele + MAP_chelsa	419,8891	4,16E+01
M_3_4	Latitude + dem_ele + range_tmp_chelsa	412,7208	1,50E+03
M_3_5	Latitude + rock_type_int + soil_thick_log	427,7891	8,01E-01
M_3_6	Latitude + rock_type_int + MAP_chelsa	415,4604	3,81E+02
M_3_7	Latitude + rock_type_int + range_tmp_chelsa	427,4303	9,58E-01
M_3_8	Latitude + soil_thick_log + MAP_chelsa	411,7137	2,48E+03
M_3_9	Latitude + soil_thick_log + range_tmp_chelsa	409,1147	9,09E+03
M_4_1	Latitude + dem_ele	405,5521	5,40E+04
M_4_10	rock_type_int + soil_thick_log	418,4105	8,71E+01
M_4_11	rock_type_int + MAP_chelsa	424,454	4,24E+00
M_4_12	rock_type_int + range_tmp_chelsa	422,2909	1,25E+01
M_4_13	soil_thick_log + MAP_chelsa	424,3785	4,41E+00
M_4_14	soil_thick_log + range_tmp_chelsa	401,2194	4,71E+05
M_4_15	MAP_chelsa + range_tmp_chelsa	404,3134	1,00E+05
M_4_2	Latitude + rock_type_int	423,1949	7,96E+00
M_4_3	Latitude + soil_thick_log	417,6212	1,29E+02
M_4_4	Latitude + MAP_chelsa	406,1876	3,93E+04
M_4_5	Latitude + range_tmp_chelsa	419,5768	4,86E+01
M_4_6	dem_ele + rock_type_int	434,9016	2,29E-02
M_4_7	dem_ele + soil_thick_log	424,7641	3,63E+00
M_4_8	dem_ele + MAP_chelsa	435,4706	1,72E-02
M_4_9	dem_ele + range_tmp_chelsa	424,6992	3,75E+00

Table S15. Linear mixed effect model specifications for genera richness displaying combination of variables tested using sampling locality as a random effect. The best model (M_4_15), incorporation latitude and mean annual precipitation) is highlighted and was chosen according to the lowest BIC and highest akaike weight.

Model	Model specification using LME, locality is used as a random effect	BICs	Akaike weights
M_1_0	Latitude + dem_ele + rock_type_int + soil_thick_log + MAP_chelsa + range_tmp_chelsa	422,6176095	4,74E-08
M_1_1	Latitude + dem_ele + rock_type_int + soil_thick_log + MAP_chelsa	424,6243616	1,74E-08
M_1_2	Latitude + dem_ele + rock_type_int + soil_thick_log + range_tmp_chelsa	419,0790102	2,78E-07
M_1_3	Latitude + dem_ele + rock_type_int + MAP_chelsa + range_tmp_chelsa	417,7927052	5,29E-07
M_1_4	Latitude + dem_ele + soil_thick_log + MAP_chelsa + range_tmp_chelsa	414,6804345	2,51E-06
M_1_5	Latitude + rock_type_int + soil_thick_log + MAP_chelsa + range_tmp_chelsa	405,1242572	0,000297926
M_1_6	dem_ele + rock_type_int + soil_thick_log + MAP_chelsa + range_tmp_chelsa	417,3134641	6,72E-07
M_2_1	Latitude + dem_ele + rock_type_int + soil_thick_log	418,8969328	3,04E-07
M_2_10	Latitude + soil_thick_log + MAP_chelsa + range_tmp_chelsa	397,1009353	0,016457014
M_2_11	dem_ele + rock_type_int + soil_thick_log + MAP_chelsa	420,9095299	1,11E-07
M_2_12	dem_ele + rock_type_int + soil_thick_log + range_tmp_chelsa	414,1289397	3,30E-06
M_2_13	dem_ele + rock_type_int + MAP_chelsa + range_tmp_chelsa	413,378034	4,81E-06
M_2_14	dem_ele + soil_thick_log + MAP_chelsa + range_tmp_chelsa	409,4868888	3,36E-05
M_2_15	rock_type_int + soil_thick_log + MAP_chelsa + range_tmp_chelsa	400,7279595	0,002683816
M_2_2	Latitude + dem_ele + rock_type_int + MAP_chelsa	418,6892497	3,38E-07
M_2_3	Latitude + dem_ele + rock_type_int + range_tmp_chelsa	418,1167498	4,50E-07
M_2_4	Latitude + dem_ele + soil_thick_log + MAP_chelsa	416,7165767	9,05E-07
M_2_5	Latitude + dem_ele + soil_thick_log + range_tmp_chelsa	411,1751553	1,45E-05
M_2_6	Latitude + dem_ele + MAP_chelsa + range_tmp_chelsa	409,8513978	2,80E-05
M_2_7	Latitude + rock_type_int + soil_thick_log + MAP_chelsa	407,0560892	0,000113401
M_2_8	Latitude + rock_type_int + soil_thick_log + range_tmp_chelsa	408,3053359	6,07E-05
M_2_9	Latitude + rock_type_int + MAP_chelsa + range_tmp_chelsa	400,433734	0,00310916
M_3_1	Latitude + dem_ele + rock_type_int	413,0202543	5,75E-06
M_3_10	Latitude + MAP_chelsa + range_tmp_chelsa	392,3762298	0,174706001
M_3_11	dem_ele + rock_type_int + soil_thick_log	418,2169625	4,28E-07
M_3_12	dem_ele + rock_type_int + MAP_chelsa	414,7865542	2,38E-06
M_3_13	dem_ele + rock_type_int + range_tmp_chelsa	417,9257209	4,95E-07
M_3_14	dem_ele + soil_thick_log + MAP_chelsa	413,4520954	4,63E-06
M_3_15	dem_ele + soil_thick_log + range_tmp_chelsa	406,3595877	0,000160642
M_3_16	dem_ele + MAP_chelsa + range_tmp_chelsa	405,7096162	0,000222331
M_3_17	rock_type_int + soil_thick_log + MAP_chelsa	406,4060312	0,000156955
M_3_18	rock_type_int + soil_thick_log + range_tmp_chelsa	401,7714867	0,001592772
M_3_19	rock_type_int + MAP_chelsa + range_tmp_chelsa	398,3985746	0,008601462
M_3_2	Latitude + dem_ele + soil_thick_log	410,5359401	1,99E-05
M_3_20	soil_thick_log + MAP_chelsa + range_tmp_chelsa	392,9100456	0,133780001
M_3_3	Latitude + dem_ele + MAP_chelsa	410,6980986	1,84E-05
M_3_4	Latitude + dem_ele + range_tmp_chelsa	410,1620367	2,40E-05
M_3_5	Latitude + rock_type_int + soil_thick_log	409,3329669	3,63E-05
M_3_6	Latitude + rock_type_int + MAP_chelsa	400,8435468	0,002533106
M_3_7	Latitude + rock_type_int + range_tmp_chelsa	416,0325034	1,27E-06
M_3_8	Latitude + soil_thick_log + MAP_chelsa	398,8873015	0,00673669
M_3_9	Latitude + soil_thick_log + range_tmp_chelsa	400,5148918	0,002985519
M_4_1	Latitude + dem_ele	404,61139	0,000385014
M_4_10	rock_type_int + soil_thick_log	402,6054636	0,001049682
M_4_11	rock_type_int + MAP_chelsa	400,2709829	0,003372749
M_4_12	rock_type_int + range_tmp_chelsa	409,6545593	3,09E-05
M_4_13	soil_thick_log + MAP_chelsa	398,7024322	0,007389081
M_4_14	soil_thick_log + range_tmp_chelsa	393,8743207	0,082604081
M_4_15	MAP_chelsa + range_tmp_chelsa	390,7481978	0,394303353
M_4_2	Latitude + rock_type_int	410,2205922	2,33E-05
M_4_3	Latitude + soil_thick_log	401,3565058	0,001960043
M_4_4	Latitude + MAP_chelsa	392,625023	0,154270565
M_4_5	Latitude + range_tmp_chelsa	408,2267192	6,32E-05
M_4_6	dem_ele + rock_type_int	415,1069188	2,02E-06
M_4_7	dem_ele + soil_thick_log	410,4611039	2,07E-05
M_4_8	dem_ele + MAP_chelsa	407,2428007	0,000103293
M_4_9	dem_ele + range_tmp_chelsa	410,4661639	2,06E-05

Table S16. Summary statistics of the fixed-effect models: (A) least squares model for genus richness and (B) generalized linear model for reproductive mode. Indicated p-values are calculated using a two-sided Wald test.

A. Least squares model genus richness				
Parameters	Value	SE	t-value	p-value
Intercept	-0.0411977	0.5056251	-0.081479	0.9352
$Slope_{soil\_thick}$	-0.4978359	0.1252874	-3.973551	0.0001
$Slope_{rangetemperature}$	0.4417225	0.1099234	4.018457	0.0001
**B. Generalized linear model reproductive mode**				
Parameters	Value	SE	t-value	p-value
Intercept	-1.2907594	0.8565084	-1.507	0.1218
$Slope_{elevation}$	0.0008667	0.0004077	2.126	0.0335*

Table S17. Variable importance for genera richness prediction in Random Forest. The most important variables given the highest %IncMSE and IncNodePurity are elevation (dem_ele), mean annual precipitation (MAP_chelsa) and latitude.

Variable	%IncMSE	IncNodePurity
Latitude	14.51069	20.364106
dem_ele	17.62021	29.888430
rock_type_int	10.34478	9.472686
soil_thick_log	11.62650	13.886717
MAP_chelsa	17.81223	23.848930
range_tmp_chelsa	13.40530	17.503840

Table S18. Variable importance for reproductive mode prediction in Random Forest. Elevation (dem_ele) is the most important variable according to the highest value of Mean Decrease accuracy and highest Mean Decrease Gini.

Variable	MeanDecreaseAccuracy	MeanDecreaseGini
dem_ele	0.6176	1.6342
MAP_chelsa	-1.2354	1.1678
Latitude	-0.4347	1.2277

Table S19. Lithological classes analyzed in the different modeling approaches from the global lithological map database GLiM.

Rock type integer	Lithological class	Implementation in code
1	Unconsolidated sediments (SU)	UncSed
2	Siliciclastic sedimentary rocks (SS)	SilRck
3	Pyroclastic rocks (PY)	PyrRck
4	Mixed sedimentary rocks (SC)	MxSRck
5	Carbonate sedimentary rocks (SM)	CarRck
6	Evaporites (EV)	EvaRck
7	Acid plutonic rocks (PA)	AciRck
8	Intermidiate volcanic rocks (VI)	InVRck
9	Basic volcanic rocks (VB)	BaVRck
10	Acidic plutonic rocks (PA)	AcPRck
11	Intermidiate plutonic rocks (PI)	InPRck
12	Basic plutonic rocks (PB)	BaPRck
13	Metamorphic rocks (MT)	MetRck
14	Water bodies (WB)	WatBod
15	Ice and Glaciers (IG)	IceGla
16	No data	nan

Table S20. Plant identification and estimation of vegetation coverage, percentage of phanerophytes, therophytes, and cactus in the sampling areas from which isolated nematodes were cultured and whose reproductive mode was assessed. The * represents a morphologically identified species without genetic data. Localities are abbreviated as follows: Eagle Point as "EPT", Aroma as "ARO", Totoral Dunes as "TDT", Altiplano as "ALT", Paposo as "PAP", and Salars are split into two sublocations "LAG" for Laguna Grande and "SLH" for Salar de Husaco.

Sampling Location	Nematode genera	Nematode reproductive mode	Plant identification	Formation	Vegetation cover over 100m2	% Phanerophytes (tree/shrub)	% Therophytes (herb)	% Cactus
LAG.22.03	Plectus	asexual	Fabiana ramulosa	High-altitude low shrubland	50	70	30	0
LAG.22.01	Acrobeloides	asexual	Fabiana ramulosa	High-altitude low shrubland	50	70	30	0
SLH.23.01	Plectus	asexual	Oxychloe andina	Salt flat	90	0	100	0
SLH.23.07	Propanagrolaimus*	asexual	Oxychloe andina	Salt flat	90	0	100	0
ALT.22.08	Panagrolaimus	asexual	Azorella compacta	High-altitude low shrubland	10	90	10	0
ALT.22.04	Panagrolaimus	asexual	Fabiana ramulosa	High-altitude low shrubland	30	70	30	0
ARO.22.31	Panagrolaimus	sexual	Browningia candelaris	Thorn forest	10	30	60	10
ARO.22.27	Eucephalobus	asexual	Dead cactus	Thorn forest	15	30	50	20
ARO.22.19	Plectus	asexual	Corryocactus brevistylus	Thorn forest	5	0	0	100
ARO.22.19	Panagrolaimus	sexual	Corryocactus brevistylus	Thorn forest	5	0	0	100
ARO.22.05	Acrobeloides	asexual	Tessaria absinthioides	Absolute desert	50	95	5	0
PAP.22.02	Panagrolaimus	sexual	Eulychnia sp (maybe)	Desert shrubland	40	50	5	45
PAP.22.29	Panagrolaimus	sexual	Solanum chilense	Desert shrubland	15	80	20	0
PAP.22.38	Panagrolaimus	sexual	Prosopis chilensis	Desert shrubland	80	80	20	0
PAP.22.39	Panagrolaimus	sexual	Schinus molle	Desert shrubland	80	80	20	0
PAP.22.17	Acrobeloides	asexual	Skytanthus acutus	Desert shrubland	10	80	20	0
TDT.23.02	Acrobeles	sexual	Dead shrub with lichens	Desert shrubland	10	90	10	
TDT.23.14	Cervidellus	sexual	Dead sticks	Desert shrubland	10	90	10	0
TDT.23.16	Acrobeles	asexual	Dead shrub with lichens	Desert shrubland	15	90	10	0

Altiplano

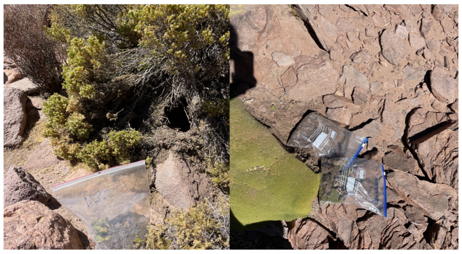

Aroma

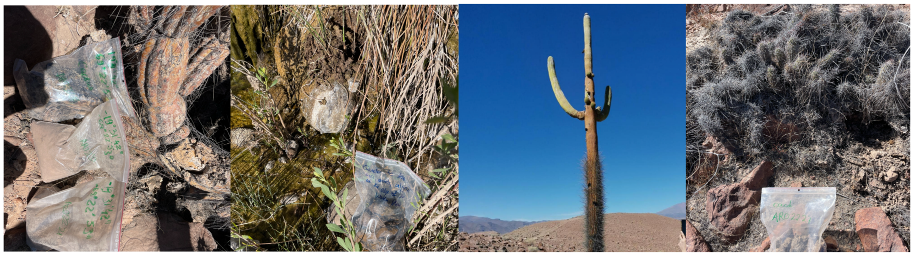

Salars

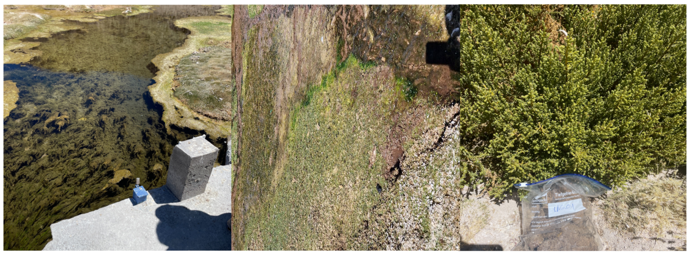

Totoral Dunes

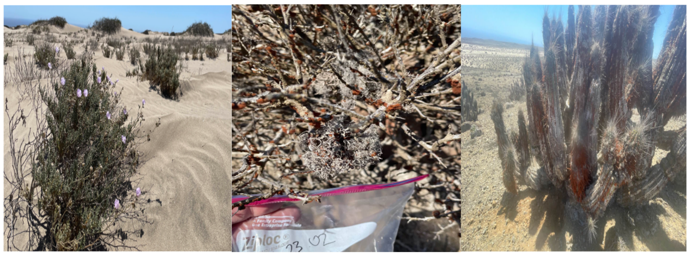

Paposo

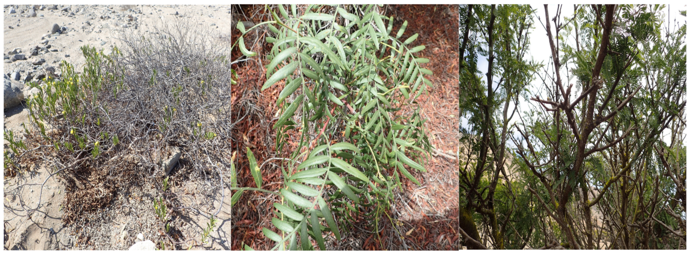

Fig. S14. Documented plants from sampled localities. These are plants from the different sampling spots from which nematodes were cultured in the lab and which (nematodes') reproductive mode was assessed. All photographs were taken by the authors.

Table S21. Model specifications for reproductive mode using GLM. the preferred model is glm1 incorporating only elevation according to lowest BIC and highest akaike weight.

Model	Model specifications for reproductive mode using GLM	BICs	Wm
glM0	dem_ele + MAP_chelsa + Latitude + range_tmp_chelsa	29,340250	0,0408556
glM1	dem_ele	24,488020	0,43632563
glM2	MAP_chelsa	26,155350	0,1895635
glM3	Latitude	26,303670	0,17601435
glM4	Latitude + dem_ele	27,367550	0,10340207
glM5	range_tmp_chelsa	30,089800	0,02731868

Table S22. Model specifications for reproductive mode using GLMM acounting for locality as a random effect. the preferred model is glm1 incorporating only elevation according to lowest BIC and highest akaike weight.

Model	Model specifications for reproductive mode using GLMM, sampling locality is used as a random effect	BICs	Wm
glM0_glmer	dem_ele + MAP_chelsa + Latitude + range_tmp_chelsa	32,23062	0,03960763
glM1_glmer	dem_ele	27,3784	0,44815349
glM2_glmer	MAP_chelsa	29,04573	0,19470216
glM3_glmer	Latitude	29,19404	0,18078573
glM4_glmer	Latitude + dem_ele	30,25792	0,10620508
glM5_glmer	range_tmp_chelsa	32,75021	0,03054591

Supplementary information for manuscript: Elevation, climate gradients, and parthenogenesis drive geographic patterns of nematode distribution in the Atacama Desert

Supplementary Note 1: correlation between geographical distance and mean annual precipitation. In order to test if the result from the modeling approach yielding mean annual temperature as one of the most important drivers for genera richness could be driven by geographical distances. To address this, we performed a mantel test between geographical distance of the localities and mean annual precipitation difference between localities (code implemented can be found on the CodeOcean capsule (10.24433/CO.6395549.v2) as Ecological estimates: Geodist_MAP. The result showed that there is no significant correlation between these two characteristics (rho= 0.1321, p= 0.24861, 719 permutations), discarding that geographical distance is correlated to precipitation, geographical distance does not reflect the precipitation patterns in our dataset.

References

1. K Gattoni, et al., 18S-NemaBase: Curated 18S rRNA Database of Nematode Sequences. *J. Nematol.* **55** (2023).
