# Supplementary material for: Geographic distribution of nematodes in the Atacama is associated with elevation, climate gradients and parthenogenesis
Source: Nat Commun. 2026 Jan 9;17:424. doi: 10.1038/s41467-025-67117-5 (PMC12796448; doi:10.1038/s41467-025-67117-5)
Supplement: Supplementary file 2 — Reporting Summary [file 41467_2025_67117_MOESM2_ESM.pdf]

nature portfolio

Corresponding author(s): Laura I. Villegas

Last updated by author(s): 04/09/2025

Reporting Summary

Nature Portfolio wishes to improve the reproducibility of the work that we publish. This form provides structure for consistency and transparency in reporting. For further information on Nature Portfolio policies, see our Editorial Policies and the Editorial Policy Checklist.

Statistics

For all statistical analyses, confirm that the following items are present in the figure legend, table legend, main text, or Methods section.

n/a	Confirmed	
☐	☒	The exact sample size (*n*) for each experimental group/condition, given as a discrete number and unit of measurement
☐	☒	A statement on whether measurements were taken from distinct samples or whether the same sample was measured repeatedly
☐	☒	The statistical test(s) used AND whether they are one- or two-sided<br>*Only common tests should be described solely by name; describe more complex techniques in the Methods section.*
☐	☒	A description of all covariates tested
☐	☒	A description of any assumptions or corrections, such as tests of normality and adjustment for multiple comparisons
☐	☒	A full description of the statistical parameters including central tendency (e.g. means) or other basic estimates (e.g. regression coefficient) AND variation (e.g. standard deviation) or associated estimates of uncertainty (e.g. confidence intervals)
☐	☒	For null hypothesis testing, the test statistic (e.g. *F*, *t*, *r*) with confidence intervals, effect sizes, degrees of freedom and *P* value noted<br>*Give P values as exact values whenever suitable.*
☒	☐	For Bayesian analysis, information on the choice of priors and Markov chain Monte Carlo settings
☐	☒	For hierarchical and complex designs, identification of the appropriate level for tests and full reporting of outcomes
☐	☒	Estimates of effect sizes (e.g. Cohen's *d*, Pearson's *r*), indicating how they were calculated

Our web collection on statistics for biologists contains articles on many of the points above.

Software and code

Policy information about availability of computer code

Data collection	The sampling data (location name, coordinates, observations) was collected using the Kobocollect and kobotoolbox platforms
Data analysis	Geneious Prime® (v. 2024.0.5), Clustal Omega (v. 1.2.3), BLAST+ (v. 2.12.0), DnaSP (v. 6.12.03) , PopART (v. 1.7), R (v 4.3.2), RStudio (2023.12.1+402), vegan (v 2.6-4),pheatmap (v. 1.0.12), cooccur package (v. 1.3), sf (v. 1.0-16), geodist (v. 0.1.0), NINJA: Nematode INdicator Joint Analysis (2014), nlme (v. 3.1), RichDEM package (v. 0.0.3), corrplot package (v. 0.92), stats package (v. 4.3.2) , factoextra package (v. 1.0.7), randomForest (v. 4.7-1.1), rsample (v. 1.2.1), caret (v. 6.0-94), pdp package (v. 0.8.2), python (v 3.10.12), Inkscape (v. 1.3.2), pandas (v. 2.1.1), xarray (v. 2023.9.0), matplotlib (v. 3.8.0), numpy (v. 1.26.0), richdem (v. 0.3.4), rioxarray (v. 0.18.1), cmcrameri (v. 1.7.1), geopandas (v. 0.14.0), shapely (v. 2.0.1), matplotlib.colors (v. 3.8.0), mpl_toolkits.axes_grid1 (v. 3.8.0)<br>. Custom codes using all the packages are deposited on github (https://github.com/lauraivillegasr/BiogeographyDesert) and are also avaiable in codeocean as capsule 6395549.<br>Code availability statement: The code implemented in this study along with the data required to run the code can be found on GitHub [https://github.com/lauraivillegasr/BiogeographyDesert] and is also available as a reproducible run on CodeOcean (10.24433/CO.6395549.v3) [https://doi.org/10.24433/CO.6395549.v3].

For manuscripts utilizing custom algorithms or software that are central to the research but not yet described in published literature, software must be made available to editors and reviewers. We strongly encourage code deposition in a community repository (e.g. GitHub). See the Nature Portfolio guidelines for submitting code & software for further information.

Data

Policy information about availability of data

All manuscripts must include a data availability statement. This statement should provide the following information, where applicable:

- Accession codes, unique identifiers, or web links for publicly available datasets
- A description of any restrictions on data availability
- For clinical datasets or third party data, please ensure that the statement adheres to our policy

The sequencing data generated in this study has been deposited in NCBI GenBank (submission: SUB14737173) and can be currently found in well as in Zenodo 10.5281/zenodo.13880073 - V6 (file name: SequencingDataBiodivAtacama.txt).. Input files used for modeling (including sampling coordinates and environmental conditions for each sampling spot) can also be found as part of the codeocean capsule and as extra files both in zenodo and the github page. The accession codes for the sequencing data are: PQ587582.1, PQ587583.1, PQ587584.1, PQ587585.1, PQ587586.1, PQ587587.1, PQ587588.1, PQ587589.1, PQ587590.1, PQ587591.1, PQ587592.1, PQ587593.1, PQ587594.1, PQ587595.1, PQ587596.1, PQ587597.1, PQ587598.1, PQ587599.1, PQ587600.1, PQ587601.1, PQ587602.1, PQ587603.1, PQ587604.1, PQ587605.1, PQ587606.1, PQ587607.1, PQ587608.1, PQ587609.1, PQ587610.1, PQ587611.1, PQ587612.1, PQ587613.1, PQ587614.1, PQ587615.1, PQ587616.1, PQ587617.1, PQ587618.1, PQ587619.1, PQ587620.1, PQ587621.1, PQ587622.1, PQ587623.1, PQ587624.1, PQ587625.1, PQ587626.1, PQ587627.1, PQ587628.1, PQ587629.1, PQ587630.1, PQ587631.1, PQ587632.1, PQ587633.1, PQ587634.1, PQ587635.1, PQ587636.1, PQ587637.1, PQ587638.1, PQ587639.1, PQ587640.1, PQ587641.1, PQ587642.1, PQ587643.1, PQ587644.1, PQ587645.1, PQ587646.1, PQ587647.1, PQ587648.1, PQ587649.1, PQ587650.1, PQ587651.1, PQ587652.1, PQ587653.1, PQ587654.1, PQ587655.1, PQ587656.1, PQ587657.1, PQ587658.1, PQ587659.1, PQ587660.1, PQ587661.1, PQ587662.1, PQ587663.1, PQ587664.1, PQ587665.1, PQ587666.1, PQ587667.1, PQ587668.1, PQ587669.1, PQ587670.1, PQ587671.1, PQ587672.1, PQ587673.1, PQ587674.1, PQ587675.1, PQ587676.1, PQ587677.1, PQ587678.1, PQ587679.1, PQ587680.1, PQ587681.1, PQ587682.1, PQ587683.1, PQ587684.1, PQ587685.1, PQ587686.1, PQ587687.1, PQ587688.1, PQ587689.1, PQ587690.1, PQ587691.1, PQ587692.1, PQ587693.1, PQ587694.1, PQ587695.1, PQ587696.1, PQ587697.1, PQ587698.1, PQ587699.1, PQ587700.1, PQ587701.1, PQ587702.1, PQ587703.1, PQ587704.1, PQ587705.1, PQ587706.1, PQ587707.1, PQ587708.1, PQ587709.1, PQ587710.1, PQ587711.1, PQ587712.1, PQ587713.1, PQ587714.1, PQ587715.1, PQ587716.1, PQ587717.1, PQ587718.1, PQ587719.1, PQ587720.1, PQ587721.1, PQ587722.1, PQ587723.1, PQ587724.1, PQ587725.1, PQ587726.1, PQ587727.1, PQ587728.1, PQ587729.1, PQ587730.1, PQ587731.1, PQ587732.1, PQ587733.1, PQ587734.1, PQ587735.1, PQ587736.1, PQ587737.1, PQ587738.1, PQ587739.1, PQ587740.1, PQ587741.1, PQ587742.1, PQ587743.1, PQ587744.1, PQ587745.1, PQ587746.1, PQ587747.1, PQ587748.1, PQ587749.1, PQ587750.1, PQ587751.1, PQ587752.1, PQ587753.1, PQ587754.1, PQ587755.1, PQ587756.1, PQ587757.1, PQ587758.1, PQ587759.1, PQ587760.1, PQ587761.1, PQ587762.1, PQ587763.1, PQ587764.1, PQ587765.1, PQ587766.1, PQ587767.1, PQ587768.1, PQ587769.1, PQ587770.1, PQ587771.1, PQ587772.1, PQ587773.1, PQ587774.1, PQ587775.1, PQ587776.1, PQ587777.1, PQ587778.1, PQ587779.1, PQ587780.1, PQ587781.1, PQ587782.1, PQ587783.1, PQ587784.1, PQ587785.1, PQ587786.1, PQ587787.1, PQ587788.1, PQ587789.1, PQ587790.1, PQ587791.1, PQ587792.1, PQ587793.1, PQ587794.1, PQ587795.1, PQ587796.1, PQ587797.1, PQ587798.1, PQ587799.1, PQ587800.1, PQ587801.1, PQ587802.1, PQ587803.1, PQ587804.1, PQ587805.1, PQ587806.1, PQ587807.1, PQ587808.1, PQ587809.1, PQ587810.1, PQ587811.1, PQ587812.1, PQ587813.1, PQ587814.1, PQ587815.1, PQ587816.1, PQ587817.1, PQ587818.1, PQ587819.1, PQ587820.1, PQ587821.1, PQ587822.1, PQ587823.1, PQ587824.1, PQ587825.1, PQ587826.1, PQ587827.1, PQ587828.1, PQ587829.1, PQ587830.1, PQ587831.1, PQ587832.1, PQ587833.1, PQ587834.1, PQ587835.1, PQ587836.1, PQ587837.1, PQ587838.1, PQ587839.1, PQ587840.1, PQ587841.1, PQ587842.1, PQ587843.1, PQ587844.1, PQ587845.1, PQ587846.1, PQ587847.1, PQ587848.1, PQ587849.1, PQ587850.1, PQ587851.1, PQ587852.1, PQ587853.1, PQ587854.1, PQ587855.1, PQ587856.1, PQ587857.1, PQ587858.1, PQ587859.1, PQ587860.1, PQ587861.1, PQ587862.1, PQ587863.1, PQ587864.1, PQ587865.1, PQ587866.1, PQ587867.1, PQ587868.1, PQ587869.1, PQ587870.1, PQ587871.1, PQ587872.1, PQ587873.1, PQ587874.1, PQ587875.1, PQ587876.1, PQ587877.1, PQ587878.1, PQ587879.1, PQ587880.1, PQ587881.1, PQ587882.1, PQ587883.1, PQ587884.1, PQ587885.1, PQ587886.1, PQ587887.1, PQ587888.1, PQ587889.1, PQ587890.1, PQ587891.1, PQ587892.1, PQ587893.1, PQ587894.1, PQ587895.1, PQ587896.1, PQ587897.1, PQ587898.1, PQ587899.1, PQ587900.1, PQ587901.1, PQ587902.1, PQ587903.1, PQ587904.1, PQ587905.1, PQ587906.1, PQ587907.1, PQ587908.1, PQ587909.1, PQ587910.1, PQ587911.1, PQ587912.1, PQ587913.1, PQ587914.1, PQ587915.1, PQ587916.1, PQ587917.1, PQ587918.1, PQ587919.1, PQ587920.1, PQ587921.1, PQ587922.1, PQ587923.1, PQ587924.1, PQ587925.1, PQ587926.1, PQ587927.1, PQ587928.1, PQ587929.1, PQ587930.1, PQ587931.1, PQ587932.1, PQ587933.1, PQ587934.1, PQ587935.1, PQ587936.1, PQ587937.1, PQ587938.1, PQ587939.1, PQ587940.1, PQ587941.1, PQ587942.1, PQ587943.1, PQ587944.1, PQ587945.1, PQ587946.1, PQ587947.1, PQ587948.1, PQ587949.1, PQ587950.1, PQ587951.1, PQ587952.1, PQ587953.1, PQ587954.1, PQ587955.1, PQ587956.1, PQ587957.1, PQ587958.1, PQ587959.1, PQ587960.1, PQ587961.1, PQ587962.1, PQ587963.1, PQ587964.1, PQ587965.1, PQ587966.1, PQ587967.1.

Data availability statement: The raw and processed data generated in this study have been deposited in the Zenodo repository database under the DOI 10.5281/zenodo.15517342 [https://doi.org/10.5281/zenodo.13880073]. The sequencing data generated in this study are deposited on GenBank with accession numbers PQ587582-PQ587967 [https://rb.gy/x6w31j].

Research involving human participants, their data, or biological material

Policy information about studies with human participants or human data. See also policy information about sex, gender (identity/presentation), and sexual orientation and race, ethnicity and racism.

Reporting on sex and gender	Not applicable.
Reporting on race, ethnicity, or other socially relevant groupings	Not applicable.
Population characteristics	Not applicable.
Recruitment	Not applicable.
Ethics oversight	Not applicable.

Note that full information on the approval of the study protocol must also be provided in the manuscript.

Field-specific reporting

Please select the one below that is the best fit for your research. If you are not sure, read the appropriate sections before making your selection.

☐ Life sciences ☐ Behavioural & social sciences ☒ Ecological, evolutionary & environmental sciences

For a reference copy of the document with all sections, see nature.com/documents/nr-reporting-summary-flat.pdf

Ecological, evolutionary & environmental sciences study design

All studies must disclose on these points even when the disclosure is negative.

Study description	In this study we collected soil samples across the Atacama Desert through a latitudinal and altitudinal gradient (coast to above 400 m.a.s.l). We covered different micro habitats including saline lakes, riverbeds, fog oases, dune systems and high altitude mountain regions. We collected nematodes from the different locations and identified them using morphological and genetic approaches. We collected environmental data using publicly available datasets like CHELSA V2.1, GLiM and the global 1-Km Gridded Thickness of Soil, Regolith, and Sedimentary Deposit Layers dataset. We aimed to answer the following questions: (1) Which biodiversity patterns can be observed in the Atacama desert at different levels (genetic, taxonomic, community, adaptative strategies)? (2) Are the diversity patterns driven by regional/local scale environmental characteristics? (3) Can we observe global trends in genera richness (diversity at community level) associated to environmental gradients?
Research sample	Samples were collected along the Atacama Desert (-69,37416667 -19,52666667 to -71,04472222 -27,79444444). Nematodes were found in 75 sample locations out of 112 sediment sample locations. All morphotypes found in the samples were sequenced (18S rRNA) using Sanger sequencing.
Sampling strategy	Samples from the upper 10-30cm of sediment were taken, nematodes were found in six defined transects (areas where no nematodes could be found were still accounted for further analysis without a specific transect name). Samples of approximately 500g were collected using a shovel and stored and transported in zip-lock bags. Nematodes were extracted from soil using seeding trays with one layer Kimberly-Clark Kimtech science precision wipes 7551. Trays were flooded from the bottom and samples were allowed to re-hydrate overnight. The water was then filtered through sieves of 80 and 120 µm. Nematodes were collected on petri dishes and examined using a Zeiss Stemi 2000 microscope. The water was sieved and nematodes were collected on petri dshes for examination and identification using a Zeiss Axioplan 2. DNA was extracted using the HotSHOT DNA extraction method, PCR for 18S rRNA was performed and assessed on a 1.5% agarose gel, amplicons were enzymatically cleaned (NEB - E1050L) and samples were submitted for sequencing .
Data collection	DNA was sequenced using Sanger sequencing at Eurofins Genomics and Genewiz. Sequences with at least 20% of high-quality bases (HQ%) were kept for further analysis. A total of 393 nematode sequences were further analyzed. Ecological data was gathered for each of the sampling coordinates using custom python script (available on github and codeocean) using the datasets CHELSA V2.1, GLiM and the global 1-Km Gridded Thickness of Soil, Regolith, and Sedimentary Deposit Layers.
Timing and spatial scale	Samples were collected from March 2022 to October 2023 during 3 sampling campaigns. Samples from the Paposo transect and are with no nematode were collected in March 2022, samples from the Aroma, Eagle Point and Altiplano transects were collected in September and October 2022, additional samples from Paposo along with samples from Salar de Huasco and the Totoral Dunes transects were collected on September and October 2023.
Data exclusions	DNA sequences with low quality were excluded to avoid biases in identification
Reproducibility	Modeling analysis of genera richness can be reproduced using the codeocean capsule, this has been previously tested.
Randomization	Several samples were taken per transect. Transect were distant to each other (minimum of 19km of distance between transects and maximum of 853 km between transects)
Blinding	The sampling order is independent from data analysis order. Data submitted to the sequencing center is assigned a numeric code with no reference to the sample location. Modeling approaches use the coordinate system for measures but not the sampling transects.

Did the study involve field work? ☒ Yes ☐ No

Field work, collection and transport

Field conditions	The sampling locations comprised several locations from the north to south of the Atacama Desert (-69,37416667 -19,52666667 to -71,04472222 -27,79444444). Samples were taken during Autumn and Spring seasons, no rainfall happened in the sampling locations in the time around the sampling campaigns. Surface temperature varied from 9 to 54 degrees celsius.
Location	The central point of the sampling transects were : Altiplano (-69.03615 -20.36505), Aroma (-69.43775 -19.58415), Eagle Point (-67.86310 -23.53000), Paposo (-70.44859 -25.06841), Salars (-68.84828 -20.35447), and Totoral Dunes (-71.04752 -27.79387). The coordinates of each sample are provided in the codeocean capsule.
Access & import/export	Samples collected in the protected area of Salar de Huasco were collected under the authorization 09/2023 from the Corporación Nacional Forestal (CONAF), samples taken outside of protected areas were also reported to the CONAF with a short summary of

	results. Soil samples were transported to Germany for exclusively for scientific research and no commercial purposes.
Disturbance	Samples were taken in areas adjacent to plants to not disturb root systems or from litter ins the Paposo locality. Samples taken in the protected area of Salar de Huasco were taken maintaining distance from Flamingos to not disturb them in the mating season.

Reporting for specific materials, systems and methods

We require information from authors about some types of materials, experimental systems and methods used in many studies. Here, indicate whether each material, system or method listed is relevant to your study. If you are not sure if a list item applies to your research, read the appropriate section before selecting a response.

Materials & experimental systems

n/a	Involved in the study
☒	☐ Antibodies
☒	☐ Eukaryotic cell lines
☒	☐ Palaeontology and archaeology
☒	☐ Animals and other organisms
☒	☐ Clinical data
☒	☐ Dual use research of concern
☒	☐ Plants

Methods

n/a	Involved in the study
☒	☐ ChIP-seq
☒	☐ Flow cytometry
☒	☐ MRI-based neuroimaging

Plants

Seed stocks	*Report on the source of all seed stocks or other plant material used. If applicable, state the seed stock centre and catalogue number. If plant specimens were collected from the field, describe the collection location, date and sampling procedures.*
Novel plant genotypes	*Describe the methods by which all novel plant genotypes were produced. This includes those generated by transgenic approaches, gene editing, chemical/radiation-based mutagenesis and hybridization. For transgenic lines, describe the transformation method, the number of independent lines analyzed and the generation upon which experiments were performed. For gene-edited lines, describe the editor used, the endogenous sequence targeted for editing, the targeting guide RNA sequence (if applicable) and how the editor was applied.*
Authentication	*Describe any authentication procedures for each seed stock used or novel genotype generated. Describe any experiments used to assess the effect of a mutation and, where applicable, how potential secondary effects (e.g. second site T-DNA insertions, mosiacism, off-target gene editing) were examined.*
